# Increasing trends of *Acinetobacter Baumannii* infections in Emilia-Romagna, Italy

**DOI:** 10.1186/1753-6561-5-S6-P292

**Published:** 2011-06-29

**Authors:** C Gagliotti, A Pan, R Buttazzi, C Capatti, E Carretto, F Pedna, M Sarti, C Venturelli, ML Moro

**Affiliations:** 1 Area Rischio Infettivo, Agenzia Sanitaria E Sociale Dell'Emilia-Romagna, Bologna, Italy; 2 Arcispedale S. Maria Nuova, Reggio Emilia, Italy; 3 AUSL Ravenna, Ravenna, Italy; 4 Nuovo Ospedale S. Agostino Estense, Italy; 5 Policlinico di Modena, Modena, Italy

## Introduction / objectives

Estimation of occurrence of *Acinetobacter baumannii* isolates from the antimicrobial surveillance system of Emilia-Romagna, Italy, 2005-2009.

## Methods

Data were collected through the laboratory based regional surveillance system. All isolates of *A. baumannii* isolates from blood, urine and respiratory samples were included in the analysis. Duplicates from the same patient/sample source within a 28 days period were excluded.

## Results

Rates of *A. baumannii* bacteraemia significantly increased between 2005 and 2009, from 0.1 to 3.2 cases/100,000 inhabitants per year. The observed increase was due to carbapenem-resistant isolates, while the number of carbapenem-susceptible isolates remained substantially stable over the study period. Importantly, the occurrence of carbapenem-resistant isolates showed a steep five-fold increase between 2008 and 2009. These isolates belonged to an epidemic strain detected in several departments of 4 hospital trusts in the Region. Similar trends were observed for urine and respiratory isolates. The total number of isolates in blood, urine and respiratory specimens, including both colonizing and infecting strains, increased from 51 in 2005 to 826 in 2009, with rates rising from 1.5 to 19.0 isolates/100,000 inhabitants per year.

## Conclusion

The temporal trends of *A. baumannii* infections are driven by carbapenem-resistant strains. A regional-wide outbreak of carbapenem-resistant *A. baumannii *infections involving 4 hospital trust was observed in Emilia-Romagna in 2009.

## Disclosure of interest

None declared.

